# miR-5100 Overexpression Inhibits Prostate Cancer Progression by Inducing Cell Cycle Arrest and Targeting E2F7

**DOI:** 10.3390/cimb46110784

**Published:** 2024-11-18

**Authors:** An Zhang, Wen Deng, Haojie Shang, Jian Wu, Yucong Zhang, Qianyuan Zhuang, Cuntai Zhang, Yuan Chen

**Affiliations:** 1 Department of Geriatrics, Tongji Hospital, Tongji Medical College, Huazhong University of Science and Technology, Wuhan 430030, China; 2 Department of Urology, Tongji Hospital, Tongji Medical College, Huazhong University of Science and Technology, No. 1095, Jiefang Avenue, Qiaokou District, Wuhan 430030, China

**Keywords:** microRNA, prostate cancer (PCa), miR-5100, E2F7, cell cycle

## Abstract

Despite advances in treatment, prostate cancer remains a leading cause of cancer-related deaths among men, highlighting the urgent need for innovative therapeutic strategies. MicroRNAs (miRNAs) have emerged as key regulatory molecules in cancer biology. In this research, we investigated the tumor-suppressive role of miR-5100 in PCa and its underlying molecular mechanism. By using RT-qPCR, we observed lower miR-5100 expression in PCa cell lines than in benign prostate cells. Functional assays demonstrated that miR-5100 overexpression significantly suppressed PCa cell proliferation, migration, and invasion. By using RNA-sequencing, we identified 446 down-regulated and 806 upregulated candidate miR-5100 target genes overrepresenting cell cycle terms. Mechanistically, E2F7 was confirmed as a direct target of miR-5100 using the reporter gene assay and RIP assay. By conducting flow cytometry analysis, cell cycle progression was blocked at the S phase. E2F7 overexpression partially mitigated the suppressive impact of miR-5100 in PCa cells. In conclusion, miR-5100 is a tumor suppressor in PCa by blocking cell cycle and targeting E2F7.

## 1. Introduction

PCa is the fifth most common cause of cancer-related mortality in men globally [1]. The intricate and diverse nature of PCa is attributed to a multitude of genetic and epigenetic modifications that influence its development and progression [2,3]. Despite advancements in early detection and treatment modalities, the precise molecular mechanisms governing the initiation and advancement of PCa remain elusive, necessitating the additional exploration of innovative biomarkers and therapeutic approaches [4,5].

MicroRNAs (miRNAs) modulate gene expression, thereby impacting various biological processes including cell proliferation, migration, and differentiation [6,7]. The dysregulated expression of miRNA has been found in various types of cancer, such as PCa [8,9,10,11]. Furthermore, miRNAs have garnered considerable attention as promising candidates for cancer diagnosis and treatment [12,13,14].

MiR-5100, which has not been extensively studied, has been found to have either tumor-suppressive or tumor-promoting properties in various types of cancer [15,16,17,18]. In the context of PCa early screening, miR-5100 has been identified as a valuable biomarker exhibiting a high degree of diagnostic sensitivity and specificity [19]. Nevertheless, its precise function in PCa remains uncertain. This research aimed to investigate the effect of miR-5100 in PCa and uncover the molecular mechanism that underlies its impact. Our data demonstrate that miR-5100 is downregulated in PCa cell lines when compared to benign prostate cells. Moreover, the excessive miR-5100 introduction exerts a suppressive impact on tumors by restraining the growth, movement, and infiltration of cells while simultaneously prompting cell cycle cessation. Our study utilized RNA-sequencing and subsequent bioinformatic analyses to uncover that dysregulated genes were notably enriched in distinct pathways, including, but not limited to, the cell cycle and DNA replication. Additionally, we detected the most significantly downregulated and upregulated cell cycle genes using qRT-PCR and further validated their expression patterns in tumor tissue and control samples from the TCGA-PRAD cohort. In PCa cells, we have effectively confirmed that miR-5100 directly targets E2F7, a transcription factor recognized for its function in modulating cell cycle progression. Furthermore, E2F7 overexpression partially counteracts the suppressive effect of miR-5100 in PCa cells. These findings underscore miR-5100’s potential as an innovative therapeutic target in the management of PCa.

## 2. Materials and Methods

### 2.1. Conditions for Cell Lines and Culturing

PC3 and DU145 prostate cancer cell lines (ATCC, Manassas, VA, USA) were grown in RPMI-1640 medium (BOSTER, Wuhan, China) containing 10% FBS. LNCAP prostate cancer cells (ATCC, Manassas, VA, USA) were cultured in H12K medium (BOSTER, Wuhan, China) containing 10% FBS. RWPE-1 cells (ATCC, Manassas, VA, USA), benign prostate cells, and 293T cells (ATCC, Manassas, VA, USA) were cultured in DMEM medium containing 5% FBS. Cells were cultured at 37 °C with 5% carbon dioxide.

### 2.2. Cell Transfection

MiR-5100 mimic, miR-NC, psiCHECK2-E2F7-MUT, psiCHECK-E2F7-WT, pcDNA3.1-E2F7, or pcDNA3.1-Empty (Genomeditech, Shanghai, China) were delivered into PC3 and DU145 cells by applying lipofectamine 3000 (Thermofisher, Waltham, MA, USA) following the manufacturer’s protocol.

### 2.3. qRT-PCR

Trizol reagent (Qiagen) was utilized to extract total RNA from the samples. To measure miR-5100 levels, 1 μg of RNA was converted into cDNA applying the miScript RT kit (Takara, Dalian, China). PCR was implemented by applying a standard SYBR Green PCR kit (Takara, Dalian, China) and miScript Primer Assays (Takara, Dalian, China), with U6 serving as the internal control. To quantify mRNA, 1 μg of RNA was transcribed in reverse using the SuperScript RT kit (Vazyme, Nanjing, China). SYBR Green kit (Vazyme, Nanjing, China) was used for PCR, with GAPDH as the internal control. The primers are listed in Table S1.

### 2.4. Cell Proliferation Assays

CCK-8 reagent (YEASON, Shanghai, China) was utilized to perform cell viability assays. The cells that underwent transfection were placed in 96-well plates with 3 × 10^3^ cells/well. After 0, 24, 48, and 72 h, the cells were exposed to 10 μL of CCK-8 for a period of 1 h. Utilizing a microplate reader (Thermofisher, Waltham, MA, USA), the measurements of absorbance were taken at 450 nm.

### 2.5. Wound-Healing Assay

Cells were distributed onto six-well plates with 3 × 10^5^ cells/well. As long as the cells had formed a single layer, a clean plastic tip was utilized for creating a mark on the cell sheet. At 10 randomly chosen positions, cells were recorded by a microscope at 0, 12, 24, and 48 h following the scratch. The width of the scratch was quantified using ImageJ to calculate the cell migration rate.

### 2.6. Cell Invasion Assays

In the invasion experiments, a total of 10 × 10^4^ cells were dropped into the top transwell chamber (BOSTER, Wuhan, China), which had been previously coated with Matrigel using serum-free medium from BD Biosciences. After incubation for 48 h, the cells on the lower chamber membrane were fixed using methanol and then 1% crystal violet, after which they were subsequently captured via microscopy and quantified.

### 2.7. Cell Cycle Assay

Cells were harvested using a Cell Cycle Staining kit (Kaiji, Suzhou, China), adhering to the established protocols. Flow cytometry analysis was performed to detect cell cycle, which was then evaluated with the Modfit 5.0 software.

### 2.8. Library Construction and Sequencing

Then, 50 nM miR-NC or miR-5100 mimics were transfected into triplicate wells of PC3 cells. Forty-eight hours after transfection, the Isolate II RNA kit (Servicebio, Wuhan, China) was applied to extract total RNA. Library preparation was performed using 1 μg of RNA for each sample. Sequencing libraries were created by purifying mRNA using magnetic beads attached with poly-T oligo. Sequencing was performed using an Novaseq 6000 platform by Aptbiotech Bioinformatics in Shanghai, China.

### 2.9. Public Dataset mRNA Data

The mRNA sequencing data from the GSE230278 dataset, consisting of 21 PCa samples and 7 benign prostate samples, were downloaded from the GEO website [20], and relative miR-5100 expression was calculated using RSEM values (RNA-seq by expectation maximization). The miR-5100 regulated cell-cycle genes were validated using the TCGA-PRAD cohort [21].

### 2.10. Dual Luciferase Reporter Gene Assays

The binding site of E2F7 and miR-5100 was obtained from the TargetScan 8.0 website. Afterward, the 3′-UTR of E2F7, which included either the binding or mutant sequences, was synthesized and then inserted into the psiCHECK-2 vector acquired from Tsingke in Beijing, China. Afterward, vectors were co-transfected into 293T cells along with either the miR-5100 mimic or miR-NC. A dual luciferase reporter assay (Vazyme, Nanjing, China) was applied for the measurement of luciferase activity. After obtaining the results, they were normalized to the activity of Renilla luciferase.

### 2.11. RNA Immunoprecipitation Assays

The RIP experiment was performed utilizing a RIP kit (Bersinbio, Guangzhou, China) following the provided instructions. In short, cells were gathered by scraping them with pre-chilled PBS. Following centrifugation, the liquid above the sediment was removed and the cells were resuspended in lysis buffer (Bersinbio, Guangzhou, China). Magnetic beads were incubated with 5 μL of anti-E2F7 antibody or rabbit IgG (Bersinbio, Guangzhou, China). Subsequently, cell lysates were introduced to the antibody-bead complexes and underwent an overnight incubation. After subjecting the immune complexes and inputting them into a series of washes, they were then subjected to elution. Subsequently, they were digested at 60 °C for 40 min. Immunoprecipitated RNA was then extracted.

### 2.12. Extraction of Proteins and Performing Western Blot Analysis

Cell lysates were used to prepare protein extracts with RIPA lysis buffer (AR0105-100) (BOSTER, Wuhan, China). Afterwards, the protein samples were isolated utilizing 4~12% SDS-PAGE gel electrophoresis (AR0047) acquired from BOSTER Biological Technology, Wuhan, China. Subsequently, they were put onto PVDF membranes (Millipore, Burlington, MA, USA). Then, the membranes were blocked and incubated with antibodies E2F7 (Abmart, Shanghai, China#TD2444; 1:1000) or β-tubulin (Abclonal, Wuhan, China#A17913; 1:5000), followed by incubation with secondary antibodies [anti-rabbit IgG (Abclonal, Wuhan, China#AS014; 1:2000) or anti-mouse IgG (Abclonal, Wuhan, China#AS003; 1:2000)]. Afterwards, the bands were observed by utilizing the ECL WB Detection System provided by Bio-Rad Laboratories located in Shanghai, China.

### 2.13. Sequencing Data Processing and Analysis

Read alignments were obtained by aligning the reads to the Human Genome (Version v101) using HISAT2 [22], and then they were assembled and merged into read coverage tables using StringTie [23]. Using featureCounts [24], the abundance of transcripts was calculated by determining the FPKM values. Through DEseq2 [25], differentially expressed genes were screened out.

### 2.14. Statistical Analysis

These results were analyzed with GraphPad Prism 8.0. Comparisons were performed statistically using either Student’s *t*-test or a one-way ANOVA, followed by Tukey’s post hoc test. Statistical significance was determined for *p*-values below 0.05, where the significance levels were indicated as * *p* < 0.05, ** *p* < 0.01, and *** *p* < 0.001.

## 3. Results

### 3.1. MiR-5100 Suppresses Proliferation, Migration, and Invasion in PCa Cells

**Figure 1:**
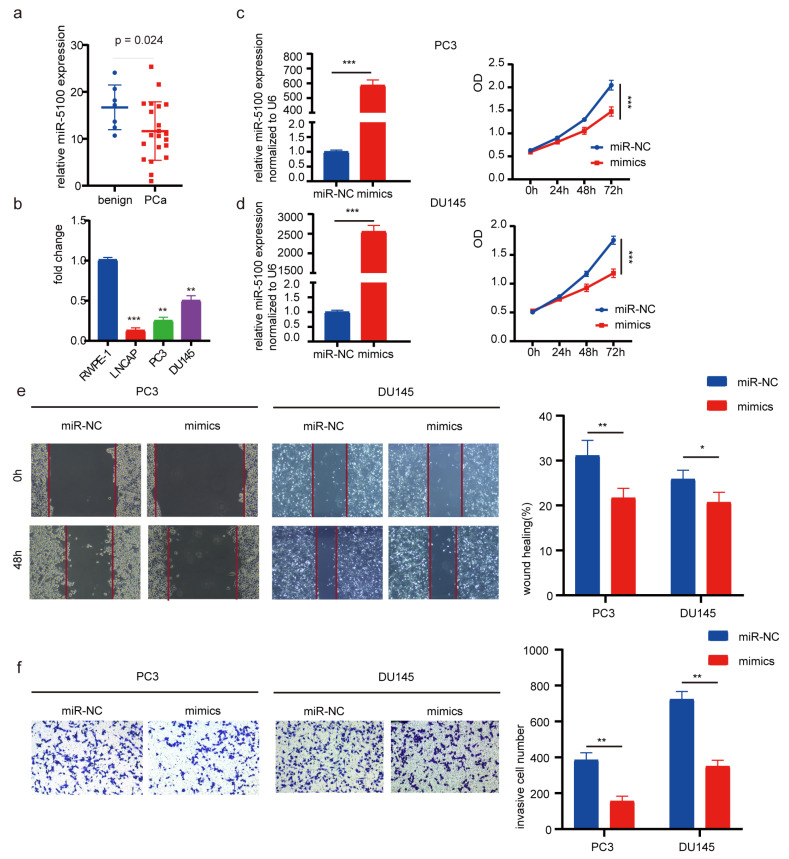
miR-5100 overexpression suppressed malignancies of PCa cells. (**a**) Expression levels of miR-5100 based on miRNA array (GEO accession: GSE230278) (*p* = 0.024). (**b**) miR-5100 expression in RWPE-1, PC3, DU145, and LNCAP cells. Values were normalized by U6 expression. (**c**,**d**) Relative expression of miR-5100 and cell viability after transfection with miR-5100 mimics. (**e**) Images and graph of scratch assay of PCa cells. (**f**) Images and graph of invaded cells. *p* < 0.05, 0.01, or 0.001 is indicated by *, **, or ***.

By analyzing the miRNA expression data in GSE230278, it was noted that miR-5100 displayed a notable decrease in PCa (log2 FC = −0.68, *p* = 0.024) (Figure 1a). Subsequently, the expression of miR-5100 was assessed in PC3, DU145, LNCAP, and RWPE-1 cells. The findings revealed that miR-5100 was lowly expressed in PCa cells (Figure 1b). Next, miR-5100 mimic and mimic-NC were delivered into PC3 and DU145 cells. The effectiveness was evaluated through RT-qPCR analysis (Figure 1c,d). The viability of PCa cells was diminished upon miR-5100 introduction during a cultivation period (Figure 1c,d). Additionally, the migration capacities of PCa cells were observed to decline after the upregulation of miR-5100, as demonstrated by scratch assays (Figure 1e). Furthermore, the invasion ability of PCa cells was found to decrease (Figure 1f). In conclusion, these findings support that miR-5100 is a suppressor of the tumorigenic ability in PCa cells.

### 3.2. Target Genes of miR-5100 Are Related to Cell Cycle

**Figure 2:**
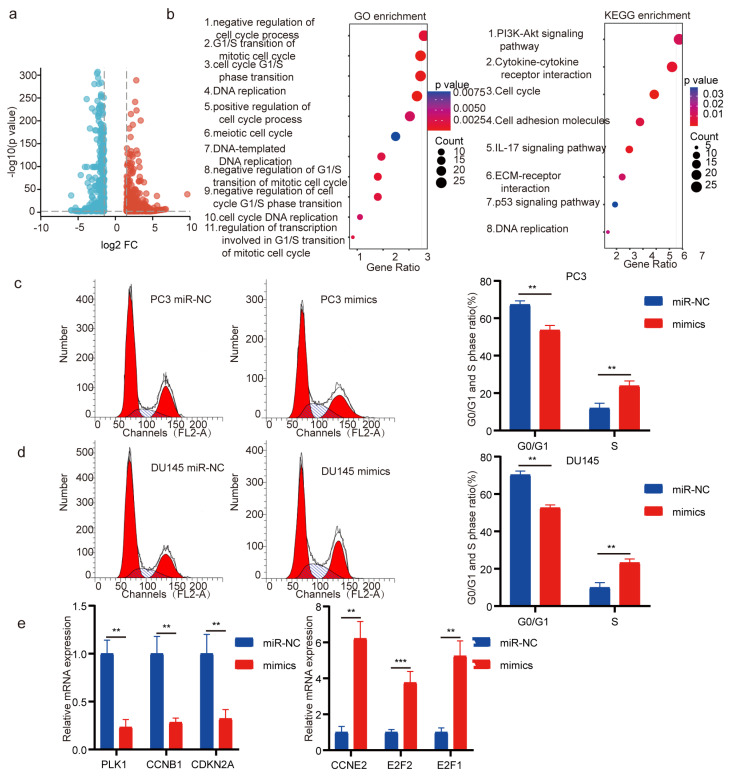
miR-5100 target genes and pathways involved in cell cycle. (**a**) Volcano plot of the differentially expressed genes. (**b**) GO enrichment analysis and KEGG pathway analysis of the differentially expressed genes. Selected top 11 altered GO terms and top 8 altered KEGG pathways. (**c**,**d**) Flow cytometry cell cycle analysis of PC3 and DU145 cells transfected with miR-NC or mimic. (**e**) mRNA expression of top 3 downregulated and top 3 upregulated cell-cycle-regulated genes normalized to GAPDH. *p* < 0.01, or 0.001 is indicated by **, or ***.

To investigate the underlying mechanism of miR-5100’s inhibitory effect on PCa cells, we conducted RNA-Seq analysis. A total of 446 down-regulated and 806 up-regulated genes were selected (Supplementary Tables S2 and S3). These genes were screened utilizing a log2 fold change exceeding 1.5 or falling below −1.5 (Figure 2a). Consequently, differentially expressed genes induced by miR-5100 were identified through transcriptome analysis. To determine the main gene networks and biological pathways controlled by miR-5100 in PC3 cells, we conducted GO and KEGG enrichment analysis with the 1252 genes that showed a differential expression. The GO analysis of these differentially expressed genes yielded 660 GO terms, with the 11 most prominent terms pertaining to cell cycle progression and DNA replication processes (Supplementary Table S4). This finding provides further evidence supporting the tumor suppressor function of miR-5100 in PCa (Figure 2b). KEGG pathway analysis revealed that ‘cell cycle’ and ‘DNA replication’ were the top altered pathways (Figure 2b, Supplementary Table S5). In general, the analysis of GO and KEGG pathways indicated that the miR-5100’s ability to inhibit tumor growth is strongly linked to its regulation of gene expression related to the cell cycle.

### 3.3. MiR-5100 Introduction Induces Cell Cycle Arrest

To explore the precise impact of miR-5100 on cell cycle progression, flow cytometry cell cycle analysis was carried out using PCa cells. S phase arrest was observed upon the introduction of miR-5100 (Figure 2c,d). Afterwards, we confirmed the expression of six key cell cycle genes that were affected by the overexpression of miR-5100 (PLK1, CCNB1, and CDKN2A were reduced; CCNE2, E2F1, and E2F2 were increased) in PC3 cells using qRT-PCR. The results were in agreement with the sequencing results (Figure 2e). Collectively, these results substantiate the notion that cell cycle modulation is a fundamental mechanism by which miR-5100 operates in PCa cells.

### 3.4. The Clinical Significance of the Cell Cycle Genes Controlled by miR-5100

**Figure 3:**
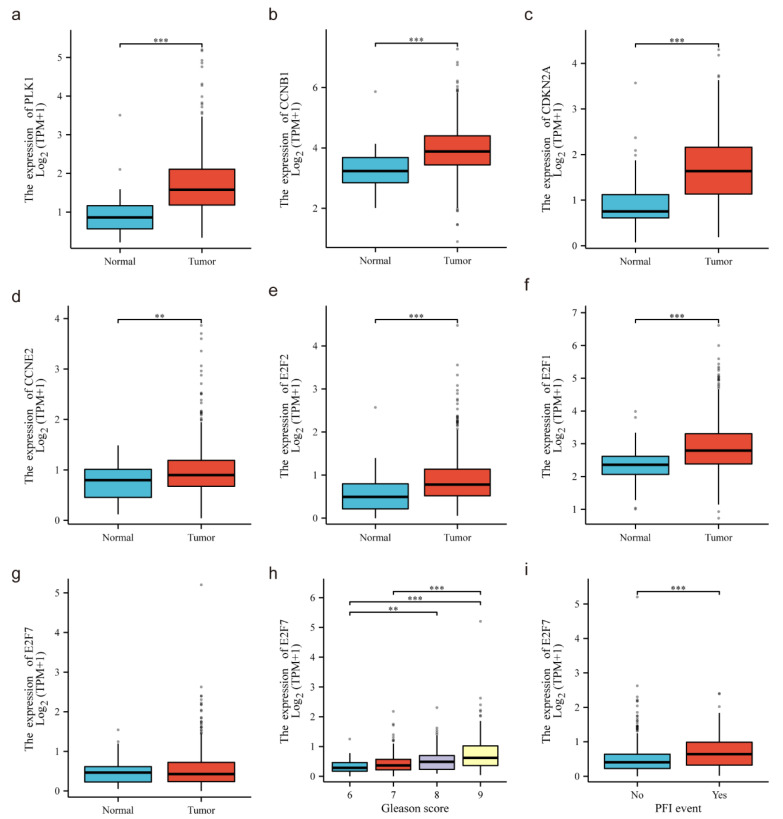
Cell-cycle-regulated genes modulated by miR-5100. (**a**–**f**) PLK1, CCNB1, CDKN2A, CCNE2, E2F2, and E2F1 expression between tumor tissues and normal tissues in PRAD dataset. (**g**) E2F7 (downregulated upon miR-5100 mimics transfection) expression between normal tissues and tumor tissues in PRAD dataset (*p* > 0.05). (**h**) E2F7 expression of tumor tissues between different Gleason scores. (**i**) E2F7 expression of tumor tissues with PFI event compared to without PFI event (*p* < 0.001). *p* < 0.01,or 0.001 is indicated by **, or ***.

To investigate the potential influence of the previously mentioned six miR-5100 targets, we analyzed their expression difference using the TCGA-PRAD dataset. We found that the down-regulated miR-5100 target genes (PLK1, CCNB1, CDKN2A) were significantly upregulated, indicating their tumor-promoting effect in PCa (Figure 3a–c). Unexpectedly, the up-regulated miR-5100 target genes (CCNE2, E2F1, E2F2) had higher levels of expression in the tumor samples (Figure 3d–f). In summary, compared to normal samples, these six genes are all highly expressed in prostate cancer samples in the TCGA-PRAD dataset.

### 3.5. E2F7 Is a Direct Target of miR-5100 in PCa Cells

**Figure 4:**
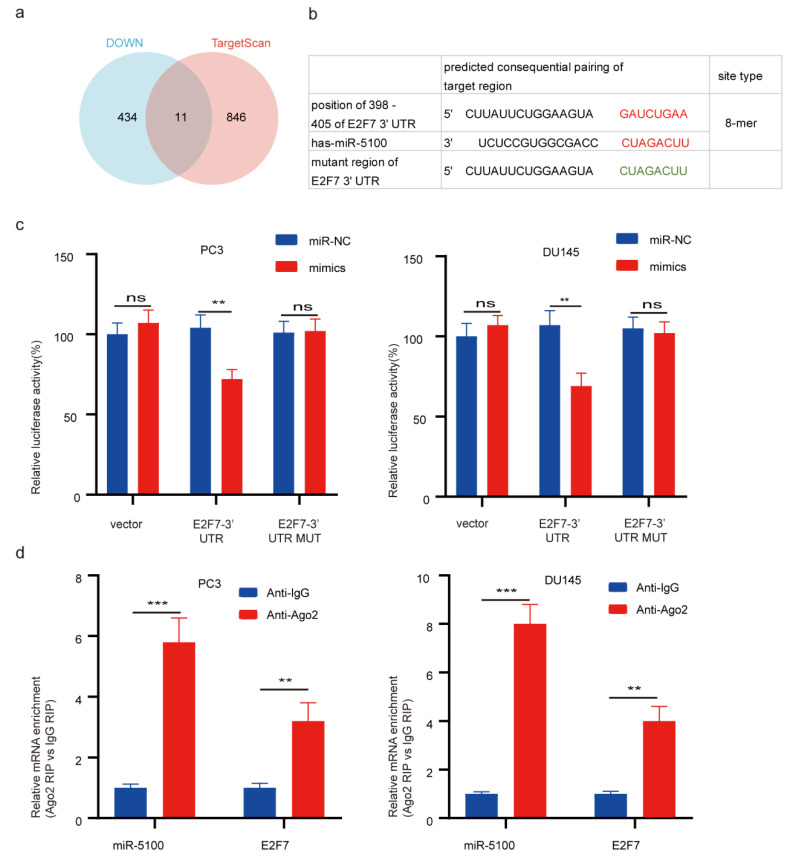
E2F7 is a direct target gene of miR-5100 in PCa cells. (**a**) Venn diagram of top 446 downregulated genes and predicted target gene set of TargetScan 8.0. (**b**) The binding site of E2F7 3′-UTR and miR-5100 seed region and the constructed mutant region of E2F7 3′-UTR. (**c**) The interaction between E2F7 3′-UTR and miR-5100 was examined by dual luciferase reporter assay. (**d**) Interaction between miR-5100 and E2F7 was confirmed by RIP assay. No statistically significance is indicated by ns; *p* < 0.01 or 0.001 is indicated by ** or ***.

To explore the direct target of miR-5100 in PCa cells, we selected 858 predicted target genes of miR-5100 from TargetScan8.0 with a cumulative weighted context++ score < −0.1 (Supplementary Table S6). By combining the 446 down-regulated genes with the 858 predicted target genes, 11 candidate genes were identified as potential direct targets of miR-5100 (Figure 4a, Supplementary Table S7). Next, by searching the 11 candidate genes in the 660 GO terms mentioned above, E2F7 was selected for further analysis since only E2F7 was involved in DNA replication and cell cycle. Furthermore, the anticipated interaction site linking miR-5100 and the 3′-UTR of E2F7 mRNA was acquired from TargetScan8.0 (Figure 4b). To ascertain this binding relationship, the wild-type and mutation of E2F7 3′-UTR (E2F7-WT and E2F7-MUT) luciferase reporter plasmids were constructed. Upon miR-5100 introduction, luciferase activity decreased in E2F7-WT cells, while no decline was detected in E2F7-MUT. This indicates that miR-5100 interacts directly with the 3′-UTR of E2F7 (Figure 4c). The RIP assay provided additional validation of the association between E2F7 messenger RNA and miR-5100. In comparison to the IgG control, the AGO2-conjugated beads showed increased expression levels of miR-5100 and E2F7 mRNA (Figure 4d). Furthermore, through the examination of the E2F7 expression pattern and its influence on patient outcomes in the TCGA-PRAD group, it was discovered that tumor samples exhibited no significant difference in E2F7 expression (Figure 3g). Moreover, high E2F7 expression was negatively associated with poor prognosis (Figure 3h,i). Collectively, these results support that miR-5100 directly targets E2F7 and indicate that E2F7 serves as an unfavorable prognostic indicator in PCa.

### 3.6. Overexpression of E2F7 Promotes Cell Proliferation and Migration and miR-5100 Rescues This Effect

**Figure 5:**
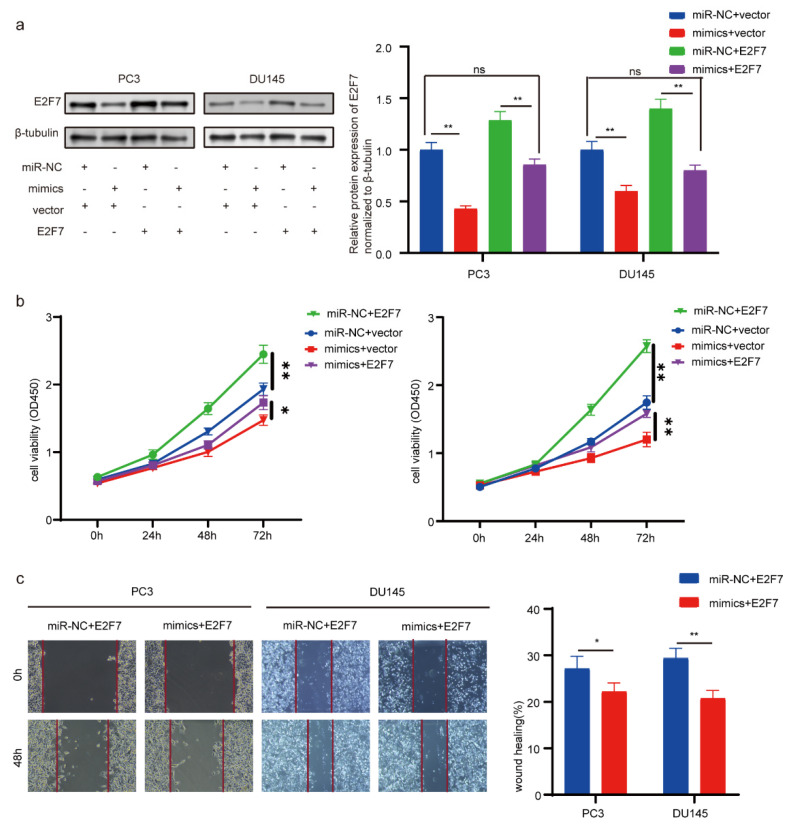
E2F7 overexpression rescue the tumor-suppressive effect of miR-5100. (**a**) Relative protein expression of E2F7. miR-NC or miR-5100 mimics and pcDNA 3.1 vector or pcDNA 3.1-E2F7 plasmid were co-transfected into PC3 or DU145 cells. (**b**) Cell viability measurement utilizing CCK-8 assay. (**c**) Images and graph of scratch assay of PCa cells. No statistically significance is indicated by ns; *p* < 0.05 or 0.01 is indicated by *, or **.

To further elucidate the extent of E2F7 in the tumor suppressive process of miR-5100, PC3 and DU145 PCa cells were transfected with plasmid E2F7 or plasmid control, together with miR-5100 mimic or miR-NC. Using Western blot, we validated that transient E2F7 overexpression enhanced E2F7 expression, and miR-5100 partially counteracted this effect (Figure 5a). Next, the overexpression of E2F7 promoted cell viability and migration in PCa cells, while miR-5100 overexpression reduced this effect (Figure 5b,c). Taken together, miR-5100 replacement significantly rescues the tumor-promoting effects of E2F7 overexpression by directly targeting E2F7 3′-UTR in PCa cells. This finding further indicates that miR-5100 could be a potent therapy in PCa.

## 4. Discussion

Dysregulated microRNAs (miRNAs) have demonstrated notable clinical significance in cancers [26,27,28]. The findings of this research provide strong evidence that miR-5100 has a tumor-suppressing role in PCa cells and also shed light on the molecular mechanism behind it. In vitro, the excessive expression of miR-5100 resulted in a notable decline in cell proliferation and migration, along with disrupted gene expression related to cell cycle regulation and DNA replication. Moreover, the dysregulation of cell-cycle-regulated genes (PLK1, CCNB1, CDKN2A, CCNE2, E2F1, and E2F2) by miR-5100 consistently resulted in upregulation in tumor tissues [29,30,31,32,33,34]. To figure out these seemingly contradictory findings, we investigated their specific roles in the cell cycle by analyzing the cell cycle pathway map in KEGG (Supplementary Figure S1). The cell cycle consists of four continuous phases (G1, S, G2, and M). In the G1 phase, CDKN2A negatively modulates DNA synthesis by deactivating CDK4, while the CCNE2-CDK2 protein complex positively regulates DNA synthesis by indirectly activating E2F1, E2F2, and E2F3. In the late G2 phase, the CCNB1-CDK1 protein complex phosphorylates and activates PLK1 to promote mitotic entry. In vitro, transient miR-5100 overexpression indirectly downregulated CDKN2A and upregulated CCNE2, E2F2, and E2F1, leading to a higher speed of G1 phase and S phase accumulation. MiR-5100 overexpression indirectly downregulated CCNB1 and PLK1, leading to a slower speed of G2/M transition. Taken together, transient miR-5100 disrupted the steady state of cell cycle in PCa cells. In vivo, upregulation of these genes synergistically accelerates every phase of the cell cycle. As soon as conditions permit, the cancer cells enter and complete the cell cycle. The available data indicate that miR-5100 has the ability to control a group of genes, either directly or indirectly, resulting in cell cycle arrest. This finding further supports the role of miR-5100 as a tumor suppressor. Furthermore, our data suggest that targeting E2F7, a cell-cycle-regulated gene modulated by miR-5100, directly may lead to significant anti-tumor effects. These results underscore the potential therapeutic implications of the miR-5100/E2F7 axis.

The controversial function of miR-5100 has been explored in various cancers. For example, miR-5100 displayed prominent upregulation in tissues of non-small-cell lung cancer, resulting in increased resistance to cisplatin by directly suppressing RAB6 expression [16,35,36]. Additionally, miR-5100 was found to be upregulated in OSCC cells, thereby facilitating cell proliferation by targeting SCAI [17]. Conversely, miR-5100 acted as a tumor suppressor in certain other malignancies. One study revealed that miR-5100 exerted a pro-apoptotic effect and hindered autophagy in gastric cells through its interaction with CAAP1 [18]. Another study observed a downregulation of miR-5100 in highly metastatic pancreatic cancer cells compared to their non-metastatic counterparts [15]. Furthermore, the heightened miR-5100 expression led to a decrease in the malignant capabilities of pancreatic tumor cells through the inhibition of PODXL [15]. Furthermore, it is important to mention that the serum miR-5100 level consistently increased in different cancers [19,37,38,39,40,41,42,43,44]. This finding suggests that serum miR-5100 holds promise as a diagnostic or prognostic indicator for these malignancies. Furthermore, miR-5100 levels were observed to be increased in exosomes obtained from hypoxic BMSCs and TAMs that lacked progranulin (PGRN-/-) [45]. The former exosomes strengthened the invasion ability of lung cancer cells through the activation of STAT3 signaling, while the latter exosomes suppressed the invasive ability of breast cancer cells via modulating CXCL12 [46].

Collectively, the impact of miR-5100 varies between promoting or suppressing tumor growth, contingent upon the particular cancer type. Additionally, the presence of miR-5100 in serum holds potential as a valuable biomarker for various cancers. Within our investigation, we found a low-level of miR-5100 expression in PCa cells. Moreover, our practical tests offered proof endorsing that miR-5100 overexpression hinders the growth and movement of PCa cells. The cell cycle analysis of flow cytometry demonstrated that miR-5100 overexpression efficiently disturbed the expression of cell cycle genes, resulting in cell cycle blockage at the S phase. This finding provides substantial evidence supporting the notion that cell cycle arrest is a crucial mechanism facilitated by miR-5100 overexpression. When considering miR-5100 as a therapeutic target, it is essential to acknowledge the complexity of miRNAs in cancer biology. While miR-5100 has shown promise as a tumor suppressor in preclinical studies, its therapeutic application must account for potential pleiotropic and off-target effects, especially in in vivo settings. To mitigate these risks, the development of precise delivery systems and rigorous in vivo testing for stability and specificity are necessary.

E2F7 modulates cell cycle genes’ expression and exerts a crucial influence on both normal and cancerous cell cycle progression [47]. Moreover, the oscillatory expression patterns of E2F transcription factor family members maintain the orderly advancement of the cell cycle, whereas the aberrant expression of these family members typically leads to cell cycle arrest [48]. Despite its general classification as a cell cycle repressor, E2F7 exhibits upregulation and oncogenic activity in diverse cancer types [49,50,51,52,53,54,55]. Multiple studies have demonstrated the tumor-promoting effect of E2F7 in PCa. Furthermore, E2F7 was found to be significantly associated with poor prognosis, tumor progression, and drug resistance in PCa [56,57,58,59]. Furthermore, the application of a nanodrug containing miR-30a-5p has demonstrated the ability to suppress malignant characteristics of ocular melanoma through targeted interaction with E2F7, as observed in both laboratory and live settings [60]. In our own investigation, we observed that the overexpression of miR-5100 effectively reduced E2F7 gene expression and counteracted the effects of E2F7 overexpression in vitro. In light of these findings, therapies aimed at targeting E2F7, such as miR-5100 replacement treatment, hold promise for reducing the growth of PCa.

## 5. Conclusions

Overall, our inquiry has revealed an unacknowledged role of miR-5100 in inhibiting tumor growth in PCa via affecting the cell cycle and targeting E2F7. These results not only contribute to our comprehension of the intricate interaction between genetic and epigenetic elements in PCa but also emphasize the therapeutic potential of targeting the miR-5100/E2F7 axis. Additional research is needed to investigate the clinical significance of miR-5100 and to assess the effectiveness of modulating the miR-5100/E2F7 axis for the treatment of PCa.

## Data Availability

The authors confirmed that the data of this study are available within the Supplementary Materials and the article. Sequencing data were uploaded to the Gene Expression Omnibus (GSE accession number = GSE267128).

## Supplementary Materials

The following supporting information can be downloaded at: https://www.mdpi.com/article/10.3390/cimb46110784/s1, Figure S1: The cell cycle pathway map in KEGG; Table S1: Specific primers used in this research; Table S2: Downregulated genes upon miR-5100 transfection; Table S3: Upregulated genes upon miR-5100 transfection; Table S4: Selected top 11 enriched GO terms; Table S5. Selected top 8 enriched KEGG pathways; Table S6: 858 predicted target genes of miR-5100 from targetScan8.0 with a cumulative weighted context++ score < −0.1; Table S7: 11potential direct target genes of miR-5100.
