# Supplementary material for: miR-5100 Overexpression Inhibits Prostate Cancer Progression by Inducing Cell Cycle Arrest and Targeting E2F7
Source: Curr Issues Mol Biol. 2024 Nov 18;46(11):13151–64. doi: 10.3390/cimb46110784 (PMC11592579; doi:10.3390/cimb46110784)

Supplementary Figure S1. cell cycle pathway map in KEGG.

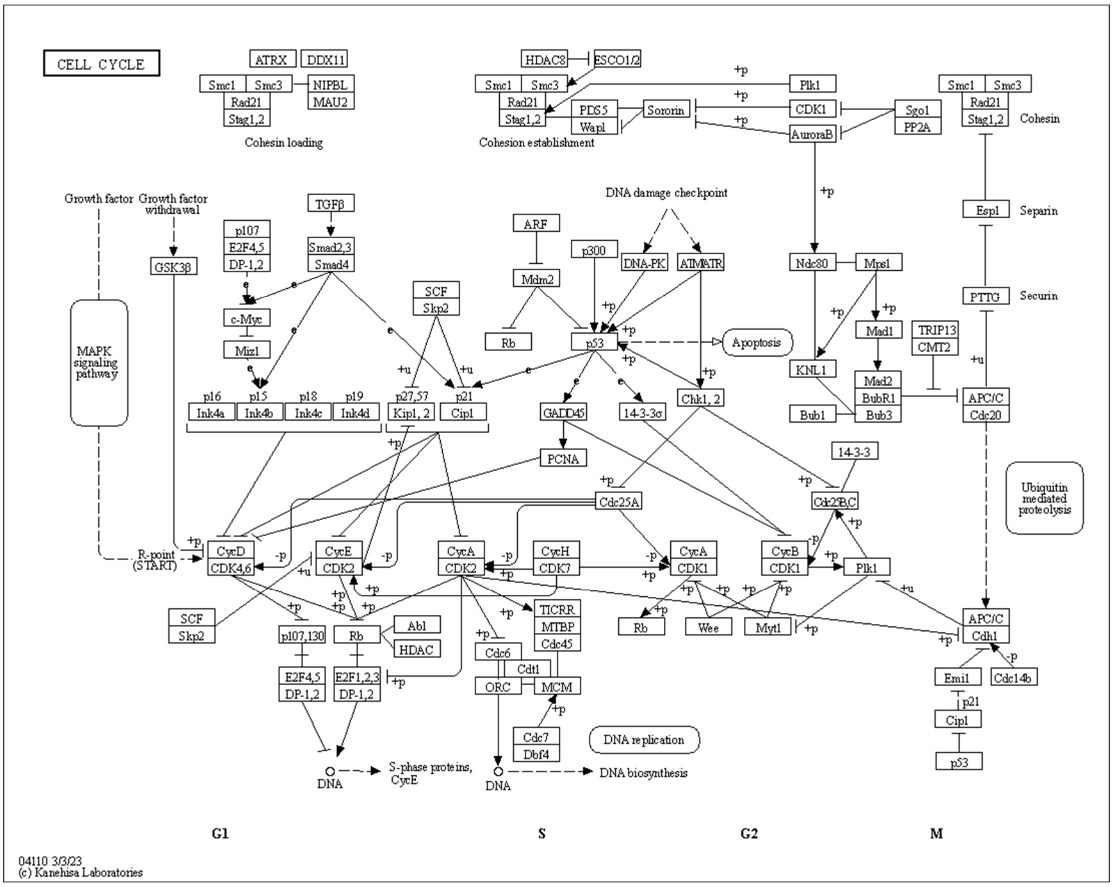

Supplement: Supplementary file 1 [file cimb-46-00784-s001.zip › Supplementary figure.pdf]
